# Adiposity, depression and anxiety: interrelationship and possible mediators

**DOI:** 10.11606/S1518-8787.2019053001119

**Published:** 2019-11-18

**Authors:** Ana Paula Gomes, Ana Luiza G. Soares, Ana M.B. Menezes, Maria Cecília Assunção, Fernando C. Wehrmeister, Laura D. Howe, Helen Gonçalves

**Affiliations:** I Universidade Federal de Pelotas. Programa de Pós-graduação em Epidemiologia. Pelotas, RS, Brasil.; II University of Bristol. Population Health Sciences. Bristol Medical School. Bristol, United Kingdom.

**Keywords:** Adiposity, Obesity, Depression, Anxiety Disorders, Measures of Association, Exposure, Risk or Outcome

## Abstract

**OBJECTIVES:**

To explore the association between adiposity, major depressive disorder and generalized anxiety disorder, and to assess the role of inflammation, diet quality and physical activity in this association.

**METHODS:**

We used data from 2,977 individuals from the 1993 Pelotas Cohort (Brazil) who attended the 18- and 22-year follow-ups. We assessed general obesity using body mass index, fat mass index, and abdominal obesity using waist circumference. Major Depressive Disorder and generalized anxiety disorder were assessed using the mini-international neuropsychiatric interview. C-reactive protein and interleukin-6 (IL-6) levels were used as a measure of inflammation; diet quality was estimated using the revised diet quality index, and physical activity was assessed by the International physical activity questionnaire (IPAQ, min/day). The association between adiposity and major depressive disorder and generalized anxiety disorder was assessed using logistic regression, and the natural indirect effect via the mediators was estimated using G-computation.

**RESULTS:**

General obesity assessed by body mass index (OR: 2.3; 95% CI:1.13; 4.85), fat mass index (OR: 2.6; 95%CI: 1.37; 4.83), and abdominal obesity (OR: 2.5; 95%CI: 1.18; 5.39) were associated with higher odds of major depressive disorder, whereas major depressive disorder was only associated with obesity assessed by body mass index (OR=1.9; 95% CI: 1.09; 3.46). Obesity and generalized anxiety disorder were not associated. C-reactive protein, diet quality and physical activity did not mediate the effect of obesity on major depressive disorder, and C-reactive protein mediated about 25% of the effect of major depressive disorder on adiposity.

**CONCLUSIONS:**

Depression, but not generalized anxiety disorder, is associated with adiposity in both directions, with a stronger evidence for the direction obesity-depression. Inflammation explains part of the effect of major depressive disorder on obesity but not the other way around. Further research should explore other mechanisms that could be involved in the association between obesity and depression.

## INTRODUCTION

Emerging adulthood (18 to 29 years old) is a critical life period of high instability, when most people have not yet set up the stable structure of an adult life. Obesity and mental disorders are common during this period^1 , 2^ , and are associated with the occurrence of several chronic diseases later in life^3 , 4^ .

Several meta-analyses of cohort studies showed a bidirectional association between major depressive disorder (MDD) (symptoms and clinical diagnosis) and obesity in adolescents and adults, and the direction from depression to obesity seems to be more common than the opposite^5 - 7^ . The effect of abdominal obesity on MDD might be higher than the effect of general obesity^8^ , and the association between depressive symptoms with changes in abdominal visceral fat has been shown to be stronger than that with changes in overall obesity^9^ . However, the evidence currently available comes from high-income countries (HIC), which limits the extrapolation of results to low- and middle-income countries (LMIC) since differences in sociocultural characteristics and lifestyle behaviors could affect such association.

The bidirectional association between anxiety and obesity has been less investigated. A prospective study with 3,134 adolescents in the United States showed that anxiety disorders predicted overweight/obesity, but not the opposite; in addition, this effect was observed only in males^10^ . Evidence regarding generalized anxiety disorder (GAD) and obesity is also weak, and the only study assessing the bidirectional relationship between body mass index (BMI) and GAD found no association, in any direction^11^ .

Several genetic, biological, social, psychological and behavioral factors could explain the interrelationship between obesity and depression/anxiety^12 , 13^ . One of the biological mechanisms is immune-inflammatory activation, as both individuals with obesity and depression have increased inflammatory markers, such as C-reactive protein (CRP), interleukin-6 (IL-6) and tumour necrosis factor-alpha (TNF-α)^12^ . Some modifiable lifestyle behaviors, such as decreased levels of physical activity and unhealthy diet, are associated with a higher risk of both obesity and depression/anxiety, and could also be involved in such association^12 , 13^ . These mechanisms, however, remain understudied.

Using data from a birth cohort in a middle-income country (Brazil), this study aimed to explore the directionality of the association between adiposity and MDD/GAD, as well as to assess the role of inflammation (CRP and IL-6 level) and lifestyle behaviors (diet quality and physical activity) in young adults.

## METHODS

### Study design and participants

Data from the 1993 Pelotas Cohort were used. In that year, all maternity hospitals in the city of Pelotas were visited daily and births were identified. Livebirths whose family lived in the urban area of the city were examined and their mothers interviewed (less than 1% of the deliveries were at home or other places). A total of 5,265 births were recorded, and 5,249 children participated in the study. Such subjects have been prospectively followed-up, and subsamples of the participants were attained until they reached the age of 11, when the full cohort was contacted. Follow-ups of the full cohort took place at 11, 15, 18 and 22 years of age. After the 18-year follow-up, interviews took place at the research clinic instead of at home. The response rate at 18 and 22 years old was 81.4% and 76.3%, respectively. Further details on study methodology are available elsewhere^14 , 15^ .

### Measures

#### Depression and anxiety

The MDD and GAD were assessed at the 18- and 22-year follow-ups using the Mini International Neuropsychiatric Interview (MINI), version 5.0^16^ . This instrument was applied by trained psychologists and was validated for the Brazilian population^17^ . Mental disorders definitions followed the DSM-5 criteria, and individuals were diagnosed with current GAD and MDD if they had presented symptoms in the 6 months and 15 days prior to the interview, respectively, in addition to moderate or severe levels of impairment. Impairment was assessed through the following question: “Thinking about your life, school, work, home, family and friends, how much have these problems impaired you? The answer options were: no impairment (0), mild impairment (1), moderate impairment (2), or severe impairment (3). Neuropsychiatric interview was not performed with deaf-mute individuals or with those with cognitive disability (n=3 at 18 years old and n=19 at 22 years old).

## Adiposity

At the 18- and 22-year follow-ups, a detailed assessment of body composition was performed, which included anthropometric measures and dual X-ray absorptiometry (DXA; GE Medical Systems Lunar). Body composition evaluation was carried out by trained and standardized assessors. For the examinations, participants wore tight-fitting gym suits (shorts and sleeveless top) and were barefoot. Exams were not performed in women who were pregnant or suspect to be pregnant and individuals with physical problems (wheelchair users and individuals with osteoarticular deformities). For DXA examination, those with weight over 120 kg or height over 192 cm (maximum device capacity), and those who had implanted metal pins, screws, plates and non-removable metallic objects (body piercings and/or chains) were not evaluated.

In this study, BMI and fat mass index (FMI) were used as indicators of total adiposity, and waist circumference (WC) as a measurement of central adiposity. Height was measured twice, using an aluminum stadiometer with accuracy of 1mm, and the mean of the two measurements was used. Weight was measured with an accuracy of 10g, using an electronic scale connected to BodPod®. The BMI was calculated dividing total weight (kg) by height squared (m^2^ ). WC was measured at the narrowest point of the waist, with a flexible tape, to the nearest 1mm. FMI was calculated by dividing total fat mass (kg), obtained by DXA, by height squared (m^2^ ).

General obesity was defined according to both BMI (≥ 30 kg/m^2^ ) and FMI (> 9 for men and > 13 for women)^18^ . Abdominal obesity was defined as WC ≥ 88 cm for women and WC ≥ 102 for men. Continuous measures of BMI, FMI and WC were also used for sensitivity analysis.

## Mediators: inflammation, diet quality and physical activity

CRP (mg/L) e IL-6 (pg/ml), diet quality, and physical activity level in leisure time or commuting, all assessed at the 18-y follow-up, were used as possible mediators.

To assess CRP and IL-6 concentration, non-fasting blood samples were collected by venipuncture, using vacutainer tubes. All samples were processed in the laboratory, stored in the same freezers at ultra-low temperature and registered in a central bio-repository. The CRP was measured by immunoturdimetric assay (Labtest Diagnóstica SA, Minas Gerais, Brazil), whereas IL-6 was measured using the Quantikine® HS Human IL-6 immunoassay kit (R&D Systems®, Inc.; Minneapolis, MN55413, USA). As CRP and IL-6 were asymmetrically distributed, they were log-transformed to obtain normalized residuals.

Total caloric intake was assessed using a food frequency questionnaire with 88 items^19^ . Diet quality was estimated using the revised diet quality index, as previously described^20^ , and analyzed as a continuous variable.

Physical activity level in leisure time and commuting (min/day) was self-reported, using the long version of the IPAQ^21^ ; hence, individuals were classified as active (> 150 minutes) or insufficiently active (< 150 minutes).

Base confounders (confounders of the association between mental disorders and adiposity)

At the perinatal visit, the following variables were assessed: sex (male/female); family income (earned by family members in the month before the interview); mother’s education level (complete years), age (years), smoking during pregnancy (yes/no), alcohol intake during pregnancy (yes/no) and pre-pregnancy BMI (calculated using self-reported weight and height); birth weight (measured in grams). Maternal smoking and alcohol intake in pregnancy were defined as any reported use of cigarettes or alcohol, respectively, at any time during pregnancy.

Maternal mental health was measured at the 11-year follow-up using the short version of the Self-Reporting Questionnaire (SRQ-20)^22^ . At the 15-year follow-up, information on the participant’s self-reported skin color (white, black/brown, yellow/indigenous), alcohol use (yes/no) and tobacco use in the previous month (yes/no) were assessed. Parenthood (yes/no) was assessed at the 18-year follow-up.

Intermediate confounders (confounders of association between each mediator and the outcome)

For mediation analysis by physical activity, diet quality at 18 years of age was considered as intermediate confounder. For the mediation analysis by diet quality, physical activity at 18 years old was considered an intermediate confounder, whereas for the mediation analysis by inflammation markers, diet quality and physical activity were both considered intermediate confounders.

## Statistical Analysis

Analyses were conducted using Stata, version 12.1 (Stata Corp., College Station, TX, USA). To assess the association between mental disorders and adiposity, logistic regression models were performed considering each direction, with and without adjustment for the potential base confounders defined above. In all analyses, those who had the outcome of interest at 18 years old were excluded to reduce the probability of reverse causality. Supplementary analyses were performed using continuous measures of adiposity, considering all participants. Standard deviations were used when adiposity was assessed as exposure, and changes in the adiposity measure between 18 and 22 years old were used when assessing it as the outcome. Tests were carried out for interactions between sex and each exposure, and no evidence was found (all p-values > 0.05).

Mediation analyses were performed using G-computation with 10,000 bootstrap replications, to estimate the natural indirect effect (NIE) ‒ mediated effect ‒ of the association between mental disorders and adiposity. Each mediator was individually included on models so that NIE corresponds to the effect by each mediator separately, instead of including it all in the same model. All mediation analyses were adjusted for the base and intermediate confounders described above, considering possible interactions between exposures and mediators. The proportion mediated was calculated as: log(OR NIE)/log(OR Total effect) ×100%.

## Ethical Considerations

All follow-ups were approved by the Medical Ethics Committee of the Federal University of Pelotas, and participants or their legal caregivers voluntarily signed an informed consent form prior to participating in the study. Individuals with high suicidal risk in the neuropsychiatric interview were referred to health services.

## RESULTS

### Sample Characteristics

This study sample size comprised 2,977 participants who had complete information on MDD/GAD and adiposity measures at both the 18-year and 22-year follow-ups. Compared to those excluded from analysis, the included were more likely to be female, to have lower socioeconomic status (lower family income and maternal education level), and to have better health indicators, e.g. lower prevalence of maternal smoking during pregnancy, common maternal mental disorders, and adolescent smoking at the age of 15 (Data not showed in Table).

**Figure 1:**
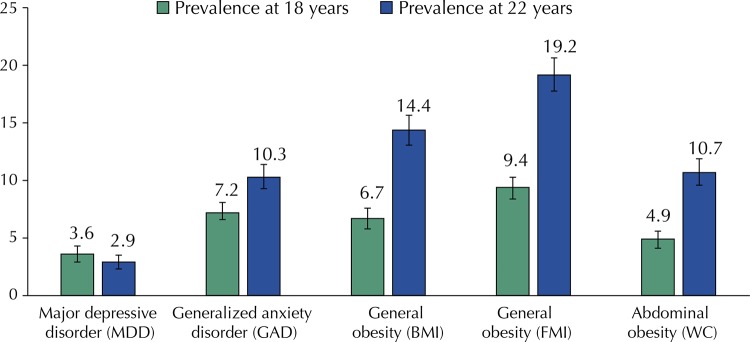
Prevalence of depression, anxiety and obesity in young adults of the 1993 Pelotas Cohort (n=2,977).

The prevalence of all obesity indicators and GAD were higher at 22 than at 18 years old, whereas the prevalence of MDD was higher at the age of 18 ( Figure 1 ).

Association of obesity at 18 years old with MDD and GAD at 22 years old

**Figure 2:**
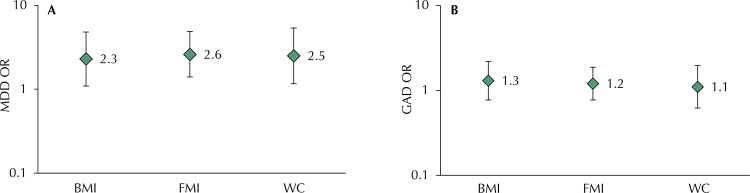
Association of obesity at 18 years of age with the incidence of major depressive disorder (A) and generalized anxiety disorder (B) between 18 and 22 years old. 1993 Pelotas Cohort, Brazil.BMI: body mass index; FMI: fat mass index; GAD: generalized anxiety disorder; MDD: major depressive disorder; WC: waist circumference

After adjustment for confounders ( Figure 2 ), all obesity measures were associated with higher odds of MDD, with a similar magnitude (OR varying from 2.3 to 2.6). No association between obesity and incidence of GAD was observed in both unadjusted or adjusted analyses. The same pattern of associations was observed using continuous measures of adiposity (data not showed in table).

Mediation of the association between adiposity and MDD

CRP, IL-6, diet quality, and physical activity did not seem to mediate the association between adiposity and MDD ( Table 1 ).

**Table 1:** Mediation by diet quality, physical activity level and inflammation markers of the association between adiposity and MDD.

Association	Mediator^¥^	Natural direct effect OR (95% CI)	Natural indirect effect OR (95% CI)	Total effect OR (95% CI)
Obesity (BMI) → MDD	Diet quality	2.76 (1.14–6.61)	0.92 (0.71–1.20)	2.55 (1.05–6.11)
	Physical activity level	2.34 (0.96–2.72)	1.14 (0.89–1.47)	2.68 (1.11–6.49)
	C-reactive protein level	1.95 (0.79–4.81)	1.05 (0.77–1.43)	2.06 (0.85–4.95)
	IL-6	2.86 (1.14–7.09)	0.81 (0.60–1.11)	2.34 (0.98–5.53)
Obesity (FMI) → MDD	Diet quality	2.86 (1.33–6.11)	0.95 (0.74–1.22)	2.72 (1.26–5.81)
	Physical activity level	2.42 (1.11–5.26)	1.14 (0.89–1.46)	2.76 (1.27–5.99)
	C-reactive protein level	2.35 (1.06–5.21)	0.95 (0.70–1.28)	2.24 (1.03–4.48)
	IL-6	3.16 (1.42–7.03)	0.80 (0.60–1.07)	2.53 (1.19–4.95)
Abdominal obesity (WC) → MDD	Diet quality	2.94 (1.19–7.24)	0.97 (0.75–1.25)	2.87 (1.14–7.17)
	Physical activity level	2.63 (1.05–6.55)	1.11 (0.88–1.35)	2.94 (1.17–6.69)
	C-reactive protein level	2.24 (0.88–5.47)	0.97 (0.71–1.32)	2.16 (0.87–5.37)
	IL-6	3.32 (1.25–8.85)	0.74 (0.53–1.05)	2.46 (0.98–6.17)

^¥^ Each mediator was analyzed separately.

BMI: body mass index; FMI: fat mass index; GAD: generalized anxiety disorder; MDD: major depressive disorder; WC: waist circumference

All analyses were adjusted for mother’s age, pre-pregnancy BMI, education level, smoking in pregnancy, alcohol use in pregnancy, and family income at birth, sex, skin color, birth weight, maternal mental health at 11 years, tobacco and alcohol use at 15 years old and parenthood at 18 years of age.

Association of MDD and GAD at 18 years old with adiposity at 22 years old

**Figure 3:**
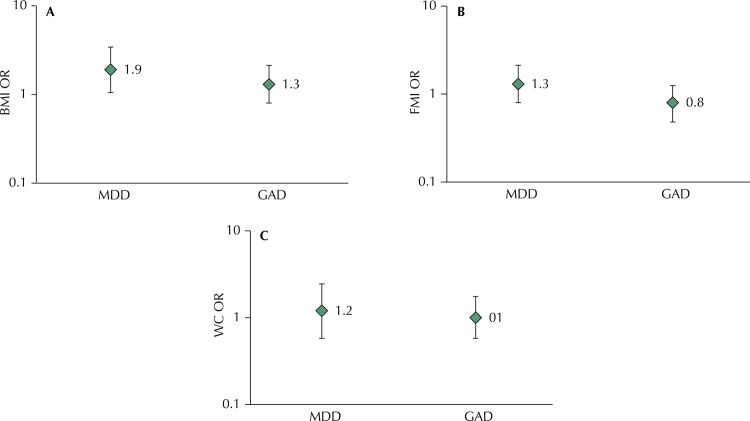
Association between major depressive disorder and generalized anxiety disorder at 18 years old, with incidence of BMI (A), FMI (B) and WC (C) between 18 and 22 years of age. BMI: body mass index; FMI: fat mass index; GAD: generalized anxiety disorder; MDD: major depressive disorder; WC: waist circumference

The MDD was associated with higher odds of general obesity regarding to BMI, in both unadjusted (OR: 2.20; 95% CI: 1.20–3.73) and adjusted analyses (OR: 1.94, 95% CI: 1.09–3.46), but no association was found with other adiposity measures ( Figure 3 ). No association was observed between GAD and any obesity measure, in unadjusted or adjusted analyses. Similar results were observed when adiposity measures were assessed as continuous measures (data not showed in table).

Mediation of the association between MDD and adiposity

The CRP concentration explained about 25% of the association between MDD and obesity measured by BMI and no evidence was found for IL-6. Diet quality and physical activity did not mediate the association between MDD and adiposity measures ( Table 2 ).

**Table 2:** Mediation analysis by diet quality, physical activity level and inflammation markers of the association between major depressive disorder and obesity (BMI).

Association	Mediator^¥^ OR (95% CI)	Natural direct effect OR (95% CI)	Natural indirect effect OR (95% CI)	Total effect OR (95% CI)
MDD → Obesity (BMI)	Diet quality	2.12 (1.11–3.67)	0.96 (0.83–1.11)	2.01 (1.12–3.67)
	Physical activity level	2.10 (1.16–3.78)	1.02 (0.88–1.18)	2.14 (1.19–3.67)
	C-reactive protein level	1.67 (0.92–2.97)	1.20 (1.01–1.42)	1.99 (1.09–3.63)
	IL-6	2.01 (1.12–3.60)	1.10 (0.94–1.29)	2.20 (1.21–4.06)
MDD → Obesity (FMI)	Diet quality	1.31 (0.68–2.51)	1.05 (0.90–1.23)	1.38 (0.72–2.63)
	Physical activity level	1.45 (0.76–2.75)	1.03 (0.89–1.20)	1.49 (0.79–2.83)
	C-reactive protein level	1.12 (0.59–2.12)	1.06 (0.90–1.25)	1.19 (0.63–2.28)
	IL-6	1.30 (0.68–2.44)	1.13 (0.96–1.32)	1.45 (0.76–2.75)
MDD → Abdominal obesity (WC)	Diet quality	1.26 (0.55–2.86)	0.97 (0.79–1.20)	1.22 (0.54–2.75)
	Physical activity level	1.32 (0.58–2.92)	1.08 (0.87–1.33)	1.42 (0.63–3.22)
	C-reactive protein level	1.12 (0.50–2.63)	1.04 (0.83–1.31)	1.16 (0.51–2.63)
	IL-6	1.30 (0.58–2.92)	1.12 (0.90–1.40)	1.46 (0.64–3.25)

^¥^ Each mediator was analyzed separately.

BMI: body mass index; FMI: fat mass index; GAD: generalized anxiety disorder; MDD: major depressive disorder; WC: waist circumference

Analyses was adjusted for the mother’s age, pre-pregnancy BMI and education level, maternal smoking in pregnancy, maternal alcohol use in pregnancy, family income at birth, sex, skin color, birth weight, maternal mental health at 11 years, tobacco and alcohol use at 15 years old and parenthood at 18 years old.

## DISCUSSION

The major findings of this study were: (1) a bidirectional association was found between MDD and obesity, even after accounting for many important, well-known confounders; (2) the effect of adiposity on MDD was stronger and more consistent than the effect of MDD on obesity; (3) GAD was not associated with obesity, and vice versa; (4) CRP, IL-6, physical activity and caloric intake did not mediate the effect of obesity on MDD, and CRP accounted for about 25% of the association between MDD and obesity (BMI).

Despite the various methods used to assess the bidirectionality of associations (longitudinal studies have used regression models, auto-regressive cross-lagged panel analyses and generalized estimating equations to access reciprocal risks), the bidirectional association between obesity and depression is consistent with the recent literature^5 - 7 , 23^ . In previous studies, the OR varies from 1.2 to 3.8 when depression leads to obesity, and from 1.1 to 5.8 when obesity leads to depression^5 , 23^ . In our study, the effect of obesity on MDD was more consistent than in the opposite direction. A recent Mendelian randomization study (using genetic variants as instrumental variables) found that higher BMI predicts depressive symptoms but not the other way around, but the authors acknowledge the weakness of genetic instruments for instrumenting depression^24^ . No difference by sex was observed in any of the associations, in accordance with previous meta-analyses^5 , 7^ . However, one should note the effect of abdominal obesity on MDD seemed to be stronger in men, even though we had little power to detect such difference.

We found no evidence of mediation by IL-6 and CRP levels on the association of obesity leading to MDD, different from a previous cohort study with English older adults, which showed that CRP levels explained about 20% of the effect of obesity on depressive symptoms over a 4-year period of follow-up^25^ . We found that about 25% of the MDD effect on obesity (BMI) was mediated by the CRP level, but no evidence of mediation was found with others adiposity measures or IL-6; Atypical MDD ‒ one distinct subtype of MDD that is characterized by reversed neurovegetative symptoms (i.e. increased appetite, leaden paralysis, and hypersomnia) ‒ has been frequently associated with high levels of inflammatory markers^26^ as well as obesity^27^ , differently than melancholic MDD. Pro-inflammatory cytokines, such as IL-6, are associated with heightened hypothalamic-pituitary-adrenal (HPA) axis activity, which can lead to elevated cortisol levels, which are known to increase appetite and preference for energy-dense food and to promote the accumulation of fat, especially in the abdominal region^12^ . As the role of CRP level on MDD and obesity association was no consistent across all adiposity measures in our study, it is important that this hypothesis would be confirmed in others longitudinal studies to rule out that this result was observed by chance.

Our study did not confirm the hypothesis that decreased levels of physical activity and consumption of low-quality diet mediated the association between obesity and MDD. We further considered other diet indicators (i.e. consumption of ultraprocessed foods and caloric intake at 18 years old), and results were similar (data not shown in this paper). A cross-sectional study using data from National Health and Nutrition Examination Surveys in the United States showed that physical activity mediated 79% of the effect of MDD on BMI in young adults, but diet quality was not involved in this association^28^ . A longitudinal study carried out with female Mexican health workers, exploring the bidirectional association between depression and body fat, showed that higher caloric intake and lower physical activity in leisure time mediated the effect of depressive symptoms on body fat, and no effects of obesity on depression were found^29^ . In our data, obesity was associated with higher physical activity, contrary to what would be expected, which could explain the lack of an indirect effect by this variable. Although counterintuitive, this association may be explained by a tendency of over-reporting physical activity among obese people, so we cannot rule out that information bias led to this result. Moreover, the exposures and mediators variables are both collected in the same follow-up and reverse causality may be present, masking the indirect effect these variables may have in the reciprocal association of adiposity and the mental disorders evaluated.

One important mechanism that could explain why depression is related to a higher risk of obesity is the use of antidepressant drugs. We intended to test such association, but the prevalence of antidepressant drug use at 18 years old in our sample was only 0.7%, and antidepressants use was not associated with obesity. Hence, it is unlikely that the effect of MDD on obesity would be completely attributable to antidepressant drugs use in our data. We acknowledge that other mechanisms linking MDD and adiposity, such as inadequate sleep, low self-esteem, body image dissatisfaction, binge eating/emotional eating, and experience of weight-related or MDD-related stigma^13^ , were not explored in this study and require further investigation.

Although plausible, the association between anxiety and adiposity has been poorly investigated. The mechanisms are very similar to those linking MDD and obesity. Biological mechanisms included the possibility of sharing a common genetic basis and HPA axis dysregulation^30^ . Weight discrimination, poorer social support and social networks could increase the risk of anxiety in obese individuals^30^ . The pressure to gain control over their weight could be distressing for obese people and increase the risk of anxiety, particularly when many attempts to lose weight have failed^30^ . Symptoms of anxiety may increase appetite and the preference for and consume of palatable “comfort foods” as a mean to alleviate their negative feelings and may, as consequence, lead to obesity^31^ .

This study did not show any association between obesity and GAD, corroborating a previous bidirectional study on this same association^11^ . Even though some studies have found a positive association between anxiety disorders and obesity (BMI)^30^ , such association might be related to specific anxiety disorder other than GAD. Future prospective studies should test the effect of other types of anxiety disorders and their association with adiposity.

Our study has several strengths. First, we used data from a birth cohort carried out in a middle-income country, where this association has been poorly investigated. We could examine directionality of effects in the same population, using the same instruments. Besides BMI, we also used different and more accurate measures of adiposity and were able to control for several relevant confounders, such as birth weight, maternal BMI and maternal common mental disorders, often not accounted for in previous studies.

Nevertheless, results of this study should also be considered in the context of several limitations. We were able to complete assessments with 56.7% of the original cohort, raising the possibility of non-response bias. Those excluded from the analysis were more likely to have lower socioeconomic status, which is associated with higher prevalence of mental disorders and obesity in our data. Therefore, we expect that associations reported here might be underestimated.

Although we excluded those with MDD and GAD at 18-year follow-up to reduce the probability of reverse causality, we cannot guarantee the cases were re-incident, as no information on previous diagnosis of MDD and GAD was available. Despite having high specificity, MINI is a screening tool and does not assess clinical diagnosis of mental disorders, so we might have misclassification error on the assessment of these disorders. However, the non-differential misclassification error is expected to underestimate associations. Finally, we were not able to distinguish between atypical and melancholic MDD, and some studies indicate that only individuals with atypical MDD have a higher risk of obesity.

This study suggests a bidirectional association between obesity and MDD. CRP level mediated about 25% of the effect of MDD on adiposity, whereas diet quality and physical activity had no indirect effect in this relationship. The GAD-obesity association was not confirmed in this study, but evidence of this association in other contexts should be investigated. Individuals with obesity and MDD could benefit from receiving a multidisciplinary treatment that includes psychological evaluation and weight gain monitoring, respectively. This could help prevent co-occurrence of these diseases and to better manage these conditions in those who already present both obesity and depression. Although we found no association with diet and physical activity in this analysis, a healthy lifestyle should be encouraged in these patients, as it could help obese individuals to lose weight and, consequently, alleviate depressive symptoms and reduce inflammatory markers that apparently can be involved in the association between MDD and obesity.
